# Comparative Analysis of Warp Function for Digital Image Correlation-Based Accurate Single-Shot 3D Shape Measurement

**DOI:** 10.3390/s18041208

**Published:** 2018-04-16

**Authors:** Xiao Yang, Xiaobo Chen, Juntong Xi

**Affiliations:** 1 School of Mechanical Engineering, Shanghai Jiao Tong University, Shanghai 200240, China; yangxiao1992@sjtu.edu.cn (X.Y.); xiaoboc@sjtu.edu.cn (X.C.); 2 Shanghai Key Laboratory of Advanced Manufacturing Environment, Shanghai 200030, China; 3 State Key Laboratory of Mechanical System and Vibration, Shanghai 200240, China

**Keywords:** single-shot 3D shape measurement, digital image correlation, warp function, inverse compositional Gauss-Newton algorithm

## Abstract

Digital image correlation (DIC)-based stereo 3D shape measurement is a kind of single-shot method, which can achieve high precision and is robust to vibration as well as environment noise. The efficiency of DIC has been greatly improved with the proposal of inverse compositional Gauss-Newton (IC-GN) operators for both first-order and second-order warp functions. Without the algorithm itself, both the registration accuracy and efficiency of DIC-based stereo matching for shapes with different complexities are closely related to the selection of warp function, subset size, and convergence criteria. Understanding the similarity and difference of the impacts of prescribed subset size and convergence criteria on first-order and second-order warp functions, and how to choose a proper warp function and set optimal subset size as well as convergence criteria for different shapes are fundamental problems in realizing efficient and accurate 3D shape measurement. In this work, we present a comparative analysis of first-order and second-order warp functions for DIC-based 3D shape measurement using IC-GN algorithm. The effects of subset size and convergence criteria of first-order and second-order warp functions on the accuracy and efficiency of DIC are comparatively examined with both simulation tests and real experiments. Reference standards for the selection of warp function for different kinds of 3D shape measurement and the setting of proper convergence criteria are recommended. The effects of subset size on the measuring precision using different warp functions are also concluded.

## 1. Introduction

Optical 3D shape measurement has become one of the research hotspots in the field of measurement due to the advantages of high precision, non-contact, and high speed, etc. Laser scanning [1,2,3], structured light [4,5], and digital image correlation (DIC) [6,7,8] are commonly used for accurate 3D shape measurement. According to previous researches [6,9], all of the three methods can achieve the same level of precision. The principle of laser scanning can be briefly summarized as: a laser line stripe plane is projected onto a measuring surface, then a laser stripe is formed and modulated by the depth of the surface. By calibrating the line stripe plane previously and recording the laser stripe by a well-calibrated camera, the 3D information along the stripe line on the surface can be characterized. For structured light measurement, coded fringe patterns are projected onto a measuring surface, the captured images are processed by relative decoding method, whereby an exact phase is computed for each pixel. The phase value is used as a measure for getting depth information of the pixel during 3D reconstruction. Laser scanning is robust to severe environment, but it needs several scans to obtain a complete shape. Structured light measurement is fast at obtaining full-field shape, which can be classified into single-shot and multiple-shot methods according to the number of projected fringe patterns. Multiple-shot structured light measurement can achieve high precision but is sensitive to vibration. Single-shot structured light measurement does not have synchronization problem between projector and camera(s) but is inaccurate at large slope or discontinuities [10]. DIC-based shape measurement is an accurate single-shot method, which is usually accompanied with speckle projection to enhance the surface characteristic, but the calculation amount is much larger than laser scanning and structured light measurement. 

The principle basis of DIC-based 3D shape measurement is binocular stereovision. The key component of 3D reconstruction by the way of stereovision is stereo matching. DIC is adopted as a region-matching algorithm to get the disparity of the same characteristic in the left (reference) image and right (target) image: it is assumed that a warp function can be used to describe the mapping relation of two local regions around the same characteristic with proper warp parameters. The warp parameters are optimized by sub-pixel registration algorithm, which is usually the most time-consuming step. The forward additive Newton-Raphson (FA-NR) algorithm is a typical iterative updating method, which is widely used with first-order [11,12] and second-order [13,14] warp functions in last decade. However, the limitation of FA-NR is that the Hessian matrix must be re-computed and inverted in each iteration, which leads to a heavy calculation burden. A more efficient algorithm called inverse compositional Gauss-Newton (IC-GN) [15] was proven to have the same accuracy as classical forward additive image alignment algorithm, but the Hessian matrix remains the same in each iteration of IC-GN [16]. Pan et al. first combined IC-GN and zero-mean normalized sum of square difference (ZNSSD) criterion in DIC with first-order warp function [17]. Since then, almost all the researches related to DIC adopted IC-GN algorithm for sub-pixel registration, which can be summed up as first-order and second-order IC-GN. First-order IC-GN is extensively used for real-time human pulse monitoring [18], real-time dynamic strain measurement [19], and 3D shape measurement [7]. It is worth noting that first-order warp function is a linear transformation, which can only characterize local translation, rotation, and uniform mapping. Therefore, Gao et al. [20] and Bai et al. [21] proposed operators for second-order IC-GN, which is effective to handle non-uniform complex mapping. Additionally, some researches have been done to study the factors that may influence the efficiency or accuracy of DIC, such as subset size [22], convergence criteria [23]. However, only first-order warp function is used in the studies. As far as we know, there is no comparative analysis about the measurement effectiveness and different characteristics of first-order and second-order warp functions until now. Therefore, it is hard to select a proper warp function and set optimal parameters according to the characteristics of different measurements. 

In this work, we present a comparative analysis of first-order and second-order warp functions for DIC-based 3D shape measurement using IC-GN algorithm. The influences of convergence criteria, subset size on the convergence efficiency and accuracy are comparatively studied by simulations and real tests. The remainder of this paper is organized as follows: The principle of DIC-based single-shot 3D shape measurement is introduced in Section 2. Experimental results and discussions are reported in Section 3. Finally, conclusions are drawn in Section 4.

## 2. Principle of DIC-Based Single-Shot 3D Shape Measurement

A single-shot stereo system, composed of two Charge Coupled Device (CCD) cameras and a digital projector, was introduced in our previous work [7]. A speckle pattern is projected onto the measuring object to enhance surface characteristics. With accurate stereo calibration and rectification [24], the same points locates on the same row of left image and right image due to the epipolar constraint. DIC can be used as a local stereo matching method to measure the disparity of the two same points that locate on the left and right images. Speckle projection-based DIC has been proven to have good performances in single-shot 3D measurement and the principle is introduced in this section [25,26]. 

### 2.1. Warp Function of DIC

The captured images of a same local region from two different angles of view have obvious difference due to rotation and deformation. By setting a reference subset in the reference image, the position and shape of the relative target subset in the target image can be described by a warp function with proper parameters. 

The first-order and second-order warp functions can be represented as:

(1) $$W_{1}\left(x,y;p_{1}\right)=\left[\begin{array}{c} x^{\prime }\\ y^{\prime }\\ 1 \end{array}\right]=\left[\begin{array}{ccc} 1+u_{x} & u_{y} & u\\ v_{x} & 1+v_{y} & v\\ 0 & 0 & 1 \end{array}\right]\left[\begin{array}{c} x\\ y\\ 1 \end{array}\right]$$

(2) $$p_{1}={\left(u,u_{x},u_{y},v,v_{x},v_{y}\right)}^{T}$$

(3) $$\Delta p_{1}={\left(\Delta u,\Delta u_{x},\Delta u_{y},\Delta v,\Delta v_{x},\Delta v_{y}\right)}^{T}$$

(4) $$W_{2}\left(x,y;p_{2}\right)=\left[\begin{array}{c} x^{\prime }\\ y^{\prime }\\ 1 \end{array}\right]=\left[\begin{array}{cccccc} \frac{1}{2}u_{xx} & u_{xy} & \frac{1}{2}u_{yy} & 1+u_{x} & u_{y} & u\\ \frac{1}{2}v_{xx} & v_{xy} & \frac{1}{2}v_{yy} & v_{x} & 1+v_{y} & v\\ 0 & 0 & 0 & 0 & 0 & 1 \end{array}\right]\left[\begin{array}{c} x^{2}\\ xy\\ y^{2}\\ x\\ y\\ 1 \end{array}\right]$$

(5) $$p_{2}={\left(u,u_{x},u_{y},u_{xx},u_{xy},u_{yy},v,v_{x},v_{y},v_{xx},v_{xy},v_{yy}\right)}^{T}$$

(6) $$\Delta p_{2}={\left(\Delta u,\Delta u_{x},\Delta u_{y},\Delta u_{xx},\Delta u_{xy},\Delta u_{yy},\Delta v,\Delta v_{x},\Delta v_{y},\Delta v_{xx},\Delta v_{xy},\Delta v_{yy}\right)}^{T}$$

where (x,y) denotes the local coordinate of the pixel in reference subset, $\left(x^{\prime },y^{\prime }\right)$ is the mapped coordinate of (x,y). $W_{1}\left(x,y;p_{1}\right)$ and $W_{2}\left(x,y;p_{2}\right)$ are the first-order and second-order warp functions with parameter vector $p_{1}$ and $p_{2}$, respectively. $\Delta p_{1}$ and $\Delta p_{2}$ denote the incremental parameter vectors. u and v denote the displacement components of center pixel of the reference subset in x direction and y direction, respectively. The other parameters are the first-order gradient components (i.e., $u_{x}$, $u_{y}$, $v_{x}$, $v_{y}$) and second-order gradient components (i.e., $u_{xx}$, $u_{xy}$, $u_{yy}$, $v_{xx}$, $v_{xy}$, $v_{yy}$). 

### 2.2. Principle of DIC-Based Stereo Matching Using IC-GN Algorithm

**Figure 1:**
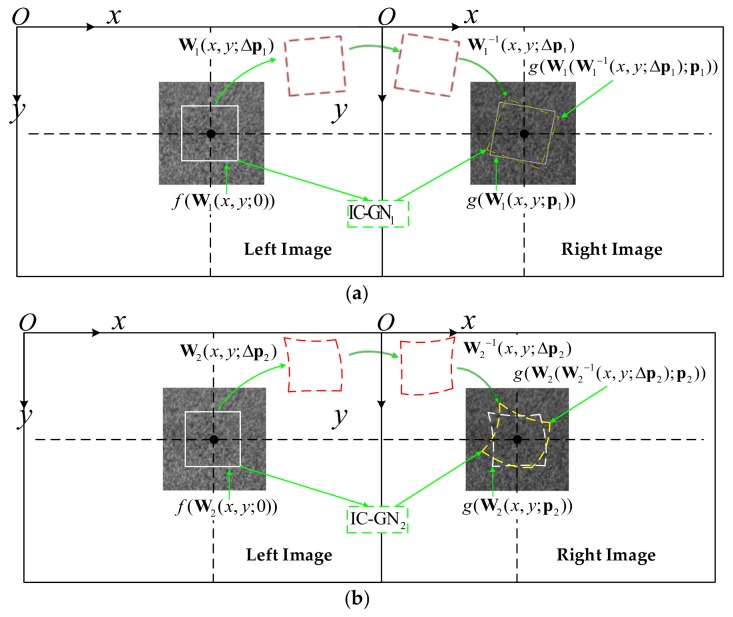
Schematic principle of DIC-based stereo matching using IC-GN algorithm: (**a**) First-order warp function; and (**b**) Second-order warp function.

Figure 1 [7] shows the schematic principle of DIC-based stereo matching using IC-GN algorithm. The first-order and second-order warp functions are adopted in Figure 1a,b, respectively. Subscript 1 and 2 are used hereinafter to distinguish the first-order and second-order IC-GN algorithms: IC-GN_1_ and IC-GN_2_. f  and g denote the gray level intensities of reference subset and target subset, respectively. A whole DIC process using IC-GN algorithm can be concluded as three steps. Firstly, compute the optimal parameter incremental vector Δp according to current p, which need to be estimated before the first iteration. The most commonly used ZNSSD criterion is employed in this step [20].

(7) $$C_{ZNSSD}\left(\Delta p\right)=\;\overset{y=M}{\underset{y=-M}{\sum }}\overset{x=M}{\underset{x=-M}{\sum }}{\left[\frac{f\left(W\left(x,y;\Delta p\right)\right)-\bar{f}}{\Delta f}-\frac{g\left(W\left(x,y;p\right)\right)-\bar{g}}{\Delta g}\right]}^{2}$$

(8) $$\Delta f=\sqrt{\sum_{y=-M}^{y=M}\sum_{x=-M}^{x=M}{\left(f\left(x,y\right)-\bar{f}\right)}^{2}},\;\Delta g=\sqrt{\sum_{y=-M}^{y=M}\sum_{x=-M}^{x=M}{\left(g\left(x^{\prime },y^{\prime }\right)-\bar{g}\right)}^{2}}$$

where $x^{\prime }$ and $y^{\prime }$ in Equation (8) are usually sub-pixel values, $g\left(x^{\prime },y^{\prime }\right)$ is calculated by B-spline interpolation [18]. $\bar{f}$ and $\bar{g}$ are the mean values of gray level intensities of the reference subset and target subset. $\bar{f}$ is constant during the iterations, while $\bar{g}$ need to be calculated in each iteration. Equation (7) can be simplified by first-order Taylor expansion with respect to Δp:

(9) $$C_{ZNSSD}\left(\Delta p\right)=\;\overset{y=M}{\underset{y=-M}{\sum }}\overset{x=M}{\underset{x=-M}{\sum }}{\left[\frac{f\left(W\left(x,y;0\right)+\nabla f\left(\frac{\partial W}{\partial p}\right)\Delta p-\bar{f}\right)}{\Delta f}-\frac{g\left(W\left(x,y;p\right)\right)-\bar{g}}{\Delta g}\right]}^{2}$$

where ∇f=(∂f/∂x,∂f/∂y) is the gray level intensity gradient in x and y directions of the reference subset. $\frac{\partial W}{\partial p}$ is the Jacobian of the warp function. For first-order and second-order warp functions, the Jacobians can be expressed respectively as:

(10) $$\frac{\partial W_{1}}{\partial p_{1}}=\left[\begin{array}{cccccc} 1 & x & y & 0 & 0 & 0\\ 0 & 0 & 0 & 1 & x & y \end{array}\right]$$

(11) $$\frac{\partial W_{2}}{\partial p_{2}}=\left[\begin{array}{cccccccccccc} 1 & x & y & \frac{1}{2}x^{2} & xy & \frac{1}{2}y^{2} & 0 & 0 & 0 & 0 & 0 & 0\\ 0 & 0 & 0 & 0 & 0 & 0 & 1 & x & y & \frac{1}{2}x^{2} & xy & \frac{1}{2}y^{2} \end{array}\right]$$

From Equation (9) Δp can be solved by least-squares method:

(12) $$\Delta p=-H^{-1}\overset{y=M}{\underset{y=-M}{\sum }}\overset{x=M}{\underset{x=-M}{\sum }}\left\{{\left[\nabla f\left(\frac{\partial W}{\partial p}\right)\right]}^{T}\left[f\left(W\left(x,y;0\right)\right)-\bar{f}-\frac{\Delta f}{\Delta g}g\left(W\left(x,y;p\right)\right)+\frac{\Delta f}{\Delta g}\bar{g}\right]\right\}$$

(13) $$H=\overset{y=M}{\underset{y=-M}{\sum }}\overset{x=M}{\underset{x=-M}{\sum }}\left\{{\left[\nabla f\left(\frac{\partial W}{\partial p}\right)\right]}^{T}\left[\nabla f\left(\frac{\partial W}{\partial p}\right)\right]\right\}$$

where H is the Hessian matrix in the IC-GN algorithm, which is constant during the iterations because ∇f and $\frac{\partial W}{\partial p}$ are independent of the target subset. 

Secondly, exert Δp on the reference subset to get the incremental warp W(x,y;Δp). Subsequently, compose current warp W(x,y;p) with the inverse incremental warp $W^{-1}\left(x,y;\Delta p\right)$ to obtain an updated warp:

(14) $$W\left(x,y;p\right)=W\left(x,y;p\right)\cdot W^{-1}\left(x,y;\Delta p\right)$$

Thirdly, repeat the above two steps with the updated p obtained by Equation (14) until preset convergence conditions have been met. In Equation (14), the warp function must be invertible. The first-order warp function can be inverted directly, while the second-order warp function need to be expanded to make it invertible [20]. 

There are usually two steps to get dense disparity map in DIC-based stereo matching, namely seed point generation and seed point propagation. Scale-invariant feature transform (SIFT) [27] is a classical feature detection method, features extracted by which is invariant to affine transformation, rotation, and scale. In this paper, SIFT-based feature detection, feature matching [28], and affine transformation are adopted to estimate initial values for p to generate seed points. The detailed procedure can be found in our previous work [7]. The initial values for $p_{1}$ can be estimated directly. For IC-GN_2_, the initial values for the second-order components (i.e., $u_{xx}$, $u_{xy}$, $u_{yy}$, $v_{xx}$, $v_{xy}$, $v_{yy}$) of $p_{2}$ are set to zeros. To improve the calculation efficiency, a fast recursive scheme [29] and reliability-guided seed point propagation [14] are utilized. Based on the disparity map, 3D reconstruction can be finished via triangulation. 

## 3. Experiments and Discussions

To conduct the comparative analysis quantitatively, two groups of experiments are investigated. In the first group, numerical simulations with two speckle images generated by computer are conducted to compare performances of first-order and second-order warp functions. In the second group, a set of experiments with different real objects are performed to evaluate the applicability and efficiency of first-order and second-order warp functions for the measurement of surfaces with different complexities. All the experiments are executed on a normal Intel(R) Core(TM) i7-4710MQ CPU 2.50 GHz laptop by C++ language with the additional library of Open Source Computer Vision (OpenCV).

In the following experiments, the modulus of the incremental displacement components Δu and Δv, $\|\Delta P_{main}\|=\sqrt{\Delta u^{2}+\Delta v^{2}}$, is used to examine the convergence. Also the optimized ZNSSD correlation coefficient is converted to zero-mean normalized cross-correlation (ZNCC) coefficient, which is equivalent to ZNSSD but more straightforward [30]. The judging conditions for the success of IC-GN are that $\left\|\Delta P_{main}\right\|$ is less than the preset convergence threshold and the optimized ZNCC coefficient is larger than 0.8, as well as the number of iterations is less than 30.

### 3.1. Comparative Analysis by Numerical Simulations 

**Figure 2:**
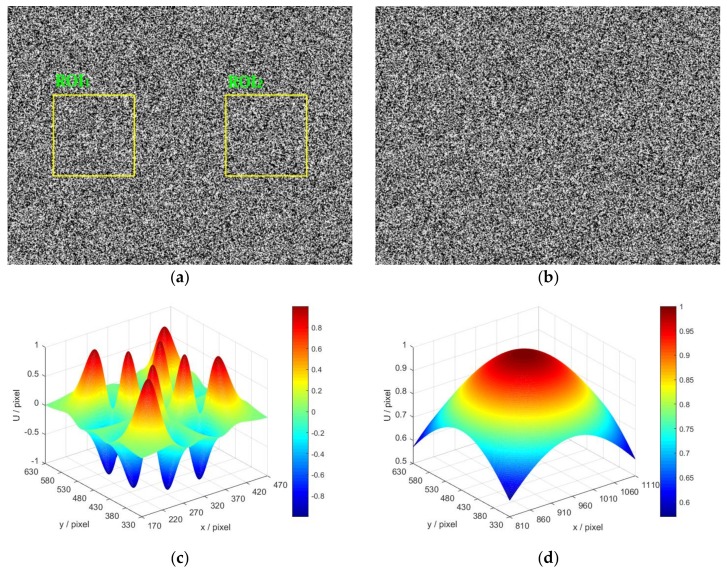
Synthetic speckle images: (**a**) Simulated reference image; (**b**) Simulated target image; (**c**) Theoretical displacements along *x*-axis of ROI_1_; and (**d**) Theoretical displacements along *x*-axis of ROI_2_.

In the following tests, a simulated image pair is equalized as a rectified stereo image pair: the displacements between the two images only occur along the along the x-axis. Therefore, the measurement of the displacements between the reference image and target image is equivalent to the process of stereo matching (getting dense disparity map) in DIC-based 3D shape measurement. As shown in Figure 2, the reference image and target images are generated by the well-known simulation algorithm proposed by Zhou [31] and widely used in previous researches [32,33,34]:

(15) $$I\left(x,y\right)=\overset{S}{\underset{k=1}{\sum }}I_{0}\exp\left(-\frac{{\left(x-x_{k}\right)}^{2}+{\left(y-y_{k}\right)}^{2}}{r^{2}}\right)$$

where I is the generated intensity of the simulated speckle image. S is the total number of speckles, r is speckle size. $\left(x_{k},y_{k}\right)$ is a randomly generated speckle position. $I_{0}$ is the peak intensity of each speckle, which is usually set to be 255. 

In Figure 2a, there are totally 150,000 randomly generated speckles in the reference image with a resolution of 1280×960 pixels, and the speckle radius is 1.2 pixels. Figure 2b is the corresponding target image generated by exerting specific displacements on the reference image:

(16) $$U\left(x,y\right)=\{\begin{array}{cc} \sin\left(2\pi E\left(x\right)/t\right)\sin\left(2\pi E\left(y\right)/t\right) & x<640\\ E\left(x\right)E\left(y\right) & x\geq 640 \end{array}$$

(17) $$E\left(x\right)=\{\begin{array}{cc} e^{-{\left(x-u_{1}\right)}^{2}/\left(2\sigma_{1}{}^{2}\right)} & x<640\\ e^{-{\left(x-u_{2}\right)}^{2}/\left(2\sigma_{2}{}^{2}\right)} & x\geq 640 \end{array}$$

(18) $$E\left(y\right)=\{\begin{array}{cc} e^{-{\left(y-v_{1}\right)}^{2}/\left(2\sigma_{1}{}^{2}\right)} & x<640\\ e^{-{\left(y-v_{2}\right)}^{2}/\left(2\sigma_{2}{}^{2}\right)} & x\geq 640 \end{array}$$

Two different forms of displacements along the x-axis are exerted on the reference image according to the displacement function U(x,y). The displacements for the left part and right part are generated by an analogous sinusoidal-Gaussian function and an analogous Gaussian function, respectively. In Figure 2a, two preset regions of interest (ROI) are marked by yellow rectangles in the left part (ROI_1_) and right part (ROI_2_). $\left(u_{1},v_{1}\right)$ and $\left(u_{2},v_{2}\right)$ are the coordinates of the center pixels of ROI_1_ and ROI_2_, which are set to be (320,480) and (960,480), respectively. σ denotes the Gaussian Root-Mean-Square (RMS) width, where $\sigma_{1}$ and $\sigma_{2}$ are set to be 50 and 200, respectively. t is the period of sinusoidal function, which is set to be 1. The displacement fields of ROI_1_ and ROI_2_ are shown in Figure 2c,d, it is obvious that the displacement field of ROI_1_ is much more complex than that of ROI_2_. 

The displacements of all the pixels in ROI_1_ and ROI_2_ are measured by IC-GN_1_ and IC-GN_2_. The measured data are analyzed statistically:

(19) $$s_{U}=\sqrt{\overset{i=N}{\underset{i=1}{\sum }}{\left(\left|U_{me}{}_{i}-U_{th}{}_{i}\right|-{\bar{e}}_{U}\right)}^{2}/\left(N-1\right)},{\bar{e}}_{U}=\frac{1}{N}\overset{i=N}{\underset{i=1}{\sum }}\left|U_{me}{}_{i}-U_{th}{}_{i}\right|$$

(20) $$RMSE_{U}=\sqrt{\overset{i=N}{\underset{i=1}{\sum }}{\left(U_{me}{}_{i}-U_{th}{}_{i}\right)}^{2}/N}$$

where ${\bar{e}}_{U}$ is the mean bias error, $s_{U}$ is the standard deviation, and $RMSE_{U}$ is the root-mean-square error (RMSE). $U_{me}{}_{i}$ and $U_{th}{}_{i}$ denote the measured and theoretical displacements along the x-axis of the sampling pixel with index i. N is the number of sampling pixels. It is necessary to state here that the influence of subset size and convergence criterion on the accuracy of IC-GN_1_ and IC-GN_2_ in different displacement fields are compared.

#### 3.1.1. Comparative Analysis with Different Subset Sizes 

**Figure 3:**
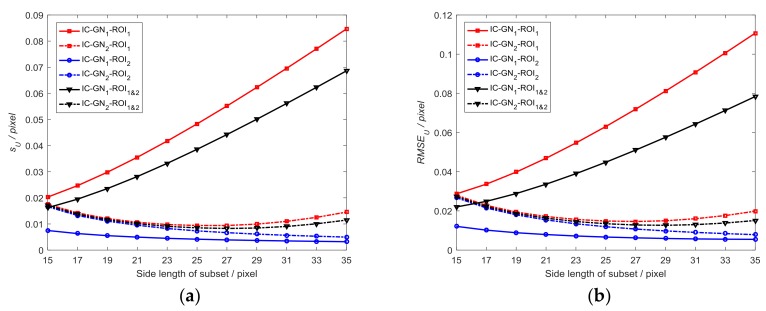
Measured displacement errors ($s_{U},RMSE_{U}$): (**a**) Standard deviation as a function of side length of subset; and (**b**) RMSE as a function of side length of subset.

**Figure 4:**
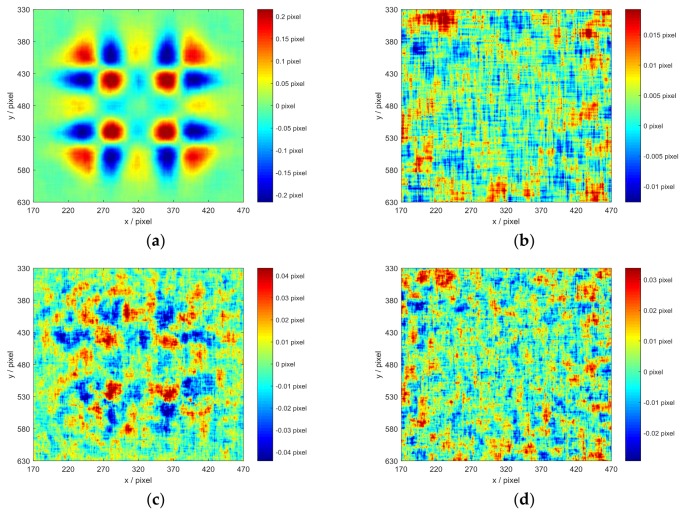
Error distribution maps with a subset size of 27×27 pixels: (**a**) Error distribution map of ROI_1_ measured by IC-GN_1_; (**b**) Error distribution map of ROI_2_ measured by IC-GN_1_; (**c**) Error distribution map of ROI_1_ measured by IC-GN_2_; and (**d**) Error distribution map of ROI_2_ measured by IC-GN_2_.

Three groups of data (namely, measured data of ROI_1_, ROI_2_, both ROI_1_ and ROI_2_) are analyzed with subset size changed from 15×15 to 35×35 pixels, where the three groups of data are denoted as ROI_1_, ROI_2_, and ROI_1&2_ hereinafter. Figure 3 shows the $s_{U}$ and $RMSE_{U}$ as a function of subset size, where the convergence threshold for $\left\|\Delta P_{main}\right\|$ is set to be 0.001. The corresponding data are listed in Table 1. To compare the characteristics of the errors measured by IC-GN_1_ and IC-GN_2_ of the two displacement fields, the error distribution maps with a specific subset size as 27×27 pixels are shown in Figure 4.

It can be easily seen in Figure 3 that for IC-GN_1_, $s_{U}$ and $RMSE_{U}$ of ROI_1_ both increase as the subset size becomes larger. However, $s_{U}$ and $RMSE_{U}$ of ROI_2_ decrease as the subset size becomes larger. For IC-GN_2_, $s_{U}$ and $RMSE_{U}$ of ROI_1_ get the minimums with the subset size of 27×27 pixels. $s_{U}$ and $RMSE_{U}$ of ROI_2_ decrease as the subset size becomes larger. For both IC-GN_1_ and IC-GN_2_, $s_{U}$ and $RMSE_{U}$ of ROI_2_ are always smaller than that of ROI_1_, which indicates that the precision of IC-GN can be reduced by complex displacement field. It should be noted that IC-GN_2_ is more accurate than IC-GN_1_ for ROI_1_. However, IC-GN_1_ is more accurate for ROI_2_ with all tested subset sizes. The errors of ROI_1&2_ are the tradeoff of errors of ROI_1_ and ROI_2_. 

The error distribution maps of ROI_1_ and ROI_2_ measured by IC-GN_1_ and IC-GN_2_ are shown in Figure 4a–d, respectively. By horizontal comparison, it is obvious that the errors of ROI_1_ measured by IC-GN_1_ and IC-GN_2_ are both mainly concentrate on the peak areas of the shape of displacement field, while the error distribution of ROI_2_ likes a random distribution. By vertical comparison, we can see that the concentrated errors in the peak areas measured by IC-GN_1_ can be suppressed by IC-GN_2_, while the errors of ROI_2_ measured by IC-GN_2_ are about double of that measured by IC-GN_1_. Therefore, it can be concluded that IC-GN_2_ is more accurate for complex displacement (disparity) field measurement, while IC-GN_1_ is more accurate for general uniform displacement (disparity) field measurement.

#### 3.1.2. Comparative Analysis with Different Convergence Criteria 

**Figure 5:**
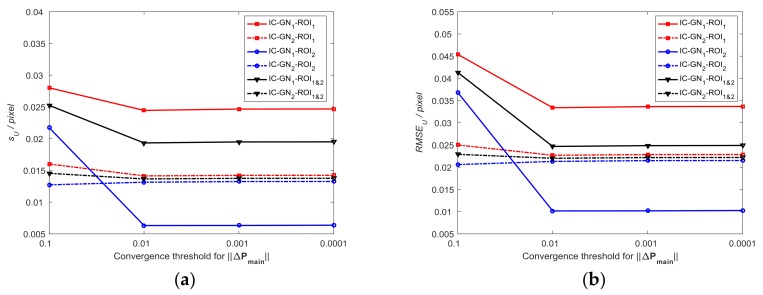
Measured displacement errors ($s_{U},RMSE_{U}$) under different convergence criteria: (**a**) Standard deviation as a function of convergence threshold for $\left\|\Delta P_{main}\right\|$; and (**b**) RMSE as a function of convergence threshold for $\left\|\Delta P_{main}\right\|$.

In Figure 3b, the curves of $RMSE_{U}$ of ROI_1&2_ measured by IC-GN_1_ and IC-GN_2_ have an intersection around the side length of subset of 17 pixels. Therefore, the subset size is set to be 17×17 pixels to compare the performances of IC-GN_1_ and IC-GN_2_ under different convergence criteria. As shown in Figure 5, the convergence threshold for $\left\|\Delta P_{main}\right\|$ is set to be 0.1, 0.01, 0.001, and 0.0001, respectively. To compare the convergence efficiency of IC-GN_1_ and IC-GN_2_ under different convergence thresholds, the average numbers of iterations (denoted as ${\bar{n}}_{itor}$) of ROI_1_, ROI_2_, and ROI_1&2_ are listed in Table 2. 

It can be concluded from Figure 5 that the same characteristic of IC-GN_1_ and IC-GN_2_ for the three groups is that the errors under the convergence thresholds of 0.01, 0.001, and 0.0001 are almost the same from each other. The difference is that the errors of IC-GN_1_ under the convergence threshold of 0.1 are significantly larger than that under the other thresholds, while the errors of IC-GN_2_ under the convergence threshold of 0.1 are slightly larger or smaller than under the other thresholds. Furthermore, $s_{U}$ and $RMSE_{U}$ of ROI_1&2_ measured by IC-GN_2_ under the convergence threshold of 0.1 are smaller than that measured by IC-GN_1_ under any one of the tested convergence thresholds. Also, it is evident in Table 2 that the preset convergence threshold directly affects the convergence efficiency. For all ROI_1_, ROI_2_, and ROI_1&2_, the average numbers of iterations of IC-GN_2_ under the convergence threshold of 0.1 are about the same as those of IC-GN_1_ under the convergence threshold of 0.01. If only ROI_1&2_ is considered, IC-GN_2_ under the convergence threshold of 0.1 is more accurate than IC-GN_1_ under the convergence threshold of 0.01. Considering both the efficiency and accuracy, conclusions can be drawn that the convergence threshold of 0.01 is the best choice for IC-GN_1_, while 0.1 is more suitable for IC-GN_2_. 

### 3.2. Comparative Anslysis by Real Tests

**Figure 6:**
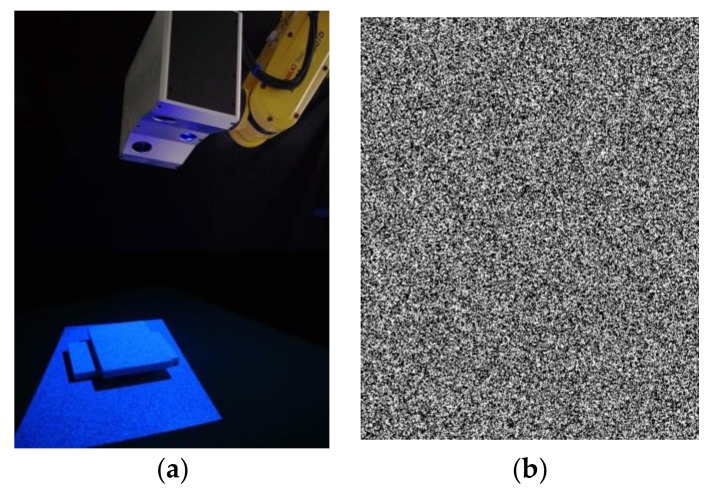
Experimental setup for real tests: (**a**) A single-shot stereo system with speckle projection; and (**b**) Projected speckle pattern.

As Shown in Figure 6a [7], a single-shot stereo system is used to perform real experiments, which is composed of two CCD cameras with a resolution of 1280 × 960 pixels (Basler acA1300-30 gm. Manufactured by Basler AG, Ahrensburg, Germany. Supplied by Shanghai Vision-Light Tech Co., Ltd. Pudong New Area, Shanghai, China), two camera lenses (Computar 8 mm 1:1.4 2/3. Manufactured by Computar^®^, Tokyo, Japan. Supplied by Shanghai Vision-Light Tech Co., Ltd. Pudong New Area, Shanghai, China), and a projector with a resolution of 1140 × 912 pixels (TI DLP LightCrafter4500. Manufactured by TEXAS INSTRUMENTS, Dallas, Texas, America. Supplied by Texas Instruments Semico…es (Shanghai) Co. Ltd. Pudong New Area, Shanghai, China). Figure 6b shows the projected speckle pattern with the same resolution as the projector: there are totally 120,000 speckles with a fixed radius of 1.2 pixels. 

**Figure 7:**
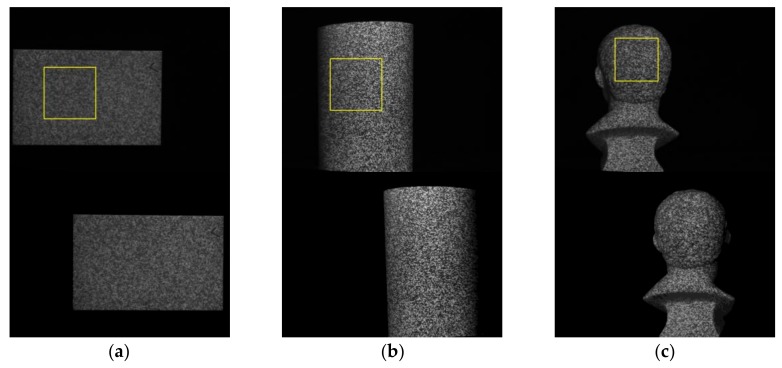
Rectified stereo image pairs for real tests, the left images are listed on the up row and the corresponding right images are listed in the bottom row: (**a**) Plane surface; (**b**) Cylinder surface; and (**c**) Back surface of a plaster head.

**Figure 8:**
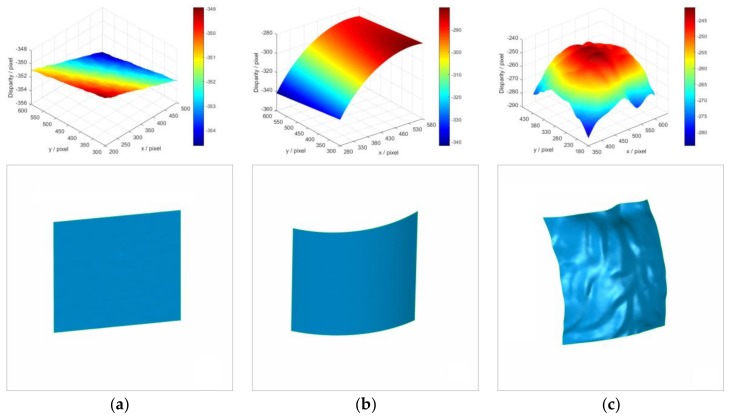
Measured disparity maps and corresponding 3D shapes, the disparity maps are listed on the up row and the corresponding 3D shapes are listed in the bottom row: (**a**) The ROI of plane; (**b**) The ROI of cylinder; and (**c**) The ROI of head.

Three objects are employed to compare the real measurement performances of IC-GN_1_ and IC-GN_2_. The measured surfaces are shown in Figure 7a–c, namely, plane surface, cylinder surface, and back surface of a plaster head (named as head hereinafter for short). The plane surface and cylinder surface are used as standard surfaces, which are measured by a Coordinate Measuring Machine (CMM (2 + (L/350) µm. Manufactured by Thome Präzision GmbH, Messel, Germany. Supplied by THOME China, Minhang District, Shanghai, China)). The 3D coordinates of the measured points are fitted into plane and cylinder surface by least square method, respectively, and the fitted results are listed in Table 3. In the calculations of real tests, a ROI is set in the left image of each rectified stereo image pair. The shape of the ROI in the head is much more complex compared to that of the plane or cylinder. The disparity maps and 3D shapes of the three ROIs are shown in Figure 8 to enable a visual comparison. In the following comparative analysis, both IC-GN_1_ and IC-GN_2_ are used for all the ROIs except for that in Figure 8, which refers to different warp function for different ROI: the ROIs in the plane and cylinder are measured by IC-GN_1_ under a convergence threshold of 0.01, and the ROI in the head is measured by IC-GN_2_ under a convergence threshold of 0.1. In addition, the subset size is set to be 27 × 27 pixels in Figure 8.

**Figure 9:**
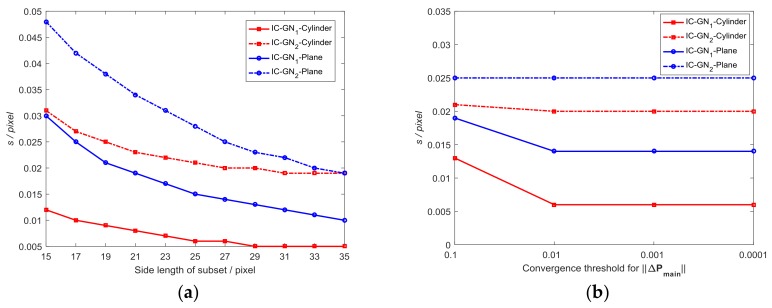
Comparisons of IC-GN_1_ and IC-GN_2_ by the standard deviations of plane fitting and cylinder surface fitting: (**a**) Comparison with the change of subset size; and (**b**) Comparison with the change of convergence threshold.

To verify the conclusions drawn by simulation tests. The pixels in each ROI are matched by IC-GN_1_ and IC-GN_2_ with the convergence threshold of 0.001, and the subset size ranges from 15 × 15 to 35 × 35 pixels. For the plane surface and cylinder surface, the standard deviation (denoted as s) of plane or cylinder surface fitting in each measurement is plotted in Figure 9a.

It can be seen that IC-GN_1_ is always more accurate than IC-GN_2_ with all tested subset sizes for both surfaces. To compare the measuring abilities of IC-GN_1_ and IC-GN_2_ for different surfaces, the statistics of matching rates with different subset size of each ROI are listed in Table 4. The matching rate is denoted as $r_{m}$, which is the ratio of number of matched pixels to the number of total pixels (denoted as $N_{pix}$) in the ROI. The matching rates of IC-GN_1_ and IC-GN_2_ are all equal or very close to 100% for the plane and cylinder surfaces. However, the matching rates of IC-GN_1_ for the ROI of head are all below 70%, while the matching rates of IC-GN_2_ are all very close to 100%. Therefore, the measurement ability of IC-GN_1_ for complex shape measurement is limited, which is almost unrelated to the change of subset size. 

**Figure 10:**
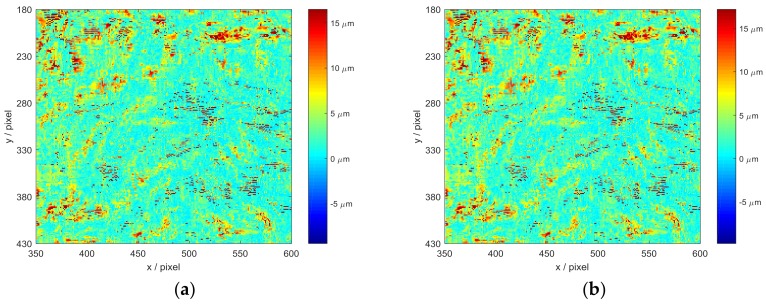
Distribution maps of spatial distance of the ROI in the head, which are measured by IC-GN_2_ under two different convergence thresholds: (**a**) Under convergence thresholds of 0.1 and 0.001; and (**b**) Under convergence thresholds of 0.01 and 0.001.

**Figure 11:**
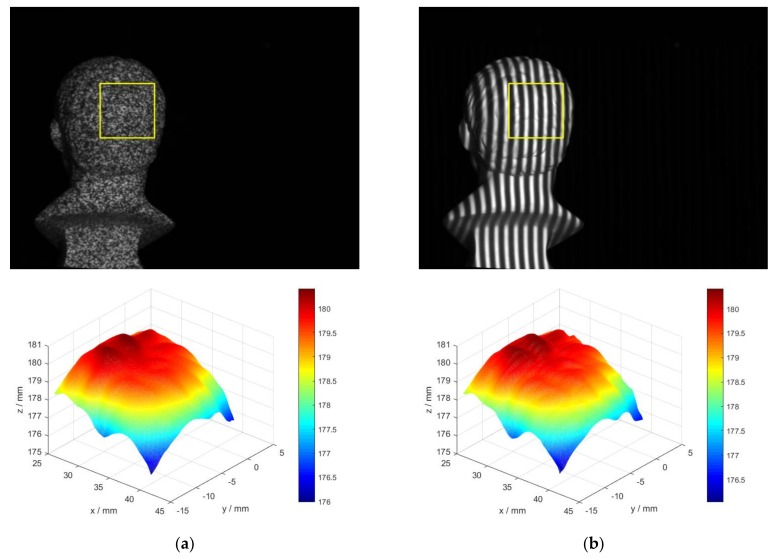
Rectified left image of head and reconstructed 3D data of the ROI marked by yellow rectangle: (**a**) Measured by IC-GN_2_ under the convergence threshold of 0.1; and (**b**) Measured by three-frequency three-step structured light.

The accuracy of IC-GN_1_ and IC-GN_2_ are also compared under different convergence thresholds with a specific subset size of 27 × 27 pixels. The standard deviations of plane fitting and cylinder surface fitting versus convergence threshold are plotted in Figure 9b. It can be seen that for IC-GN_1_, only the differences of standard deviations under convergence thresholds of 0.1 and 0.01 are relevant. For IC-GN_2_, the standard deviations are almost the same under different convergence thresholds. As shown in Figure 10, the 3D data of the ROI of head measured by IC-GN_2_ under two different convergence thresholds are compared. That means for every pixel in the ROI that has been matched, the spatial distance of the corresponding two 3D points reconstructed under the two convergence thresholds are calculated. The distance distribution maps by comparison of convergence threshold of 0.1 to 0.01 and 0.001 are shown in Figure 10a,b, respectively. Furthermore, comparison of shapes measured by IC-GN_2_ and structured light of the head are shown in Figure 11.

It needs to declare that the distance values for unmatched pixels are set to be zeros in Figure 10. There is no significant difference between Figure 10a,b; the corresponding standard deviations are 4.318 μm and 4.496 μm. To further verify the measurement effectiveness of IC-GN_2_ under the threshold of 0.1, the head is measured at the same position by both IC-GN_2_ and three-frequency three-step structured light using the same system. The same ROI is set in the rectified left images of DIC measurement and structured light measurement, and the shapes of the ROI measured by IC-GN_2_ and structured light are shown in Figure 11a,b, respectively. For every pixel in the ROI, the spatial distance of the two 3D coordinates measured by IC-GN_2_ and structured light is calculated. The standard deviation of all the calculated distance values is 0.023 mm, which is in the same level of precision of the above plane fitting and cylinder surface fitting. Therefore, conclusions can be drawn from the above comparisons that the convergence threshold of 0.01 is suitable for IC-GN_1_, while 0.1 is recommended for IC-GN_2_. The conclusions are consistent with that drawn in the simulation tests. 

## 4. Conclusions

In this paper, a comparative analysis of first-order and second-order warp functions for DIC-based stereo 3D shape measurement is presented. Both simulation tests and real experiments with different objects are performed to compare the impacts of subset size and convergence criteria on the measuring ability, efficiency, and precision by IC-GN using first-order and second-order warp functions. Conclusions are summarized as follows:

(1) The first-order warp function is more suitable for surfaces with a shape of flat or small curvature, such as plane, cylinder, and flat Gaussian surface, etc. Under the same convergence criteria, IC-GN_1_ is always more efficient and accurate than IC-GN_2_ with all tested subset sizes.
(2) The second-order warp function is more suitable for surfaces with a complex shape or large curvature, such as the tested back surface of head and analogous sinusoidal-Gaussian surface, etc. IC-GN_1_ is not capable or accurate enough for such kind of 3D shape measurement; the matching rate of tested ROI of head is under 70% with any of the tested subset size.
(3) The convergence thresholds for IC-GN_1_ and IC-GN_2_ are recommended to be that the variation of the modulus of incremental displacement vector is less than 0.01 pixel, and 0.1 pixel, respectively. Both the recommended convergence thresholds can achieve considerable measurement precision compared to smaller thresholds according to the simulation tests and real experiments.

## Figures and Tables

**Table 1:** Comparison of measured displacement errors with different subset sizes (SS) by IC-GN_1_ (1st) and IC-GN_2_ (2nd) of ROI_1_, ROI_2_, and ROI_1&2_ (unit: pixel).

SS	$s_{U}$-ROI_1_		$s_{U}$-ROI_2_		$s_{U}$-ROI_1&2_		$RMSE_{U}$-ROI_1_		$RMSE_{U}$-ROI_2_		$RMSE_{U}$-ROI_1&2_	
	1st	2nd	1st	2nd	1st	2nd	1st	2nd	1st	2nd	1st	2nd
15	0.02029	0.01755	0.00749	0.01661	0.01621	0.01709	0.02869	0.02800	0.01211	0.02673	0.02202	0.02738
17	0.02467	0.01422	0.00632	0.01324	0.01949	0.01375	0.03365	0.02284	0.01018	0.02149	0.02486	0.02218
19	0.02981	0.01207	0.00552	0.01108	0.02354	0.01159	0.03982	0.01942	0.00886	0.01800	0.02884	0.01872
21	0.03553	0.01065	0.00496	0.00952	0.02815	0.01012	0.04688	0.01713	0.00793	0.01540	0.03362	0.01629
23	0.04173	0.00977	0.00450	0.00826	0.03319	0.00908	0.05468	0.01563	0.00719	0.01342	0.03900	0.01457
25	0.04830	0.00941	0.00416	0.00736	0.03858	0.00851	0.06307	0.01484	0.00665	0.01192	0.04485	0.01346
27	0.05517	0.00941	0.00389	0.00667	0.04422	0.00827	0.07194	0.01457	0.00625	0.01073	0.05106	0.01280
29	0.06228	0.00994	0.00369	0.00612	0.05009	0.00844	0.08120	0.01497	0.00592	0.00978	0.05757	0.01264
31	0.06960	0.01099	0.00350	0.00567	0.05615	0.00904	0.09079	0.01601	0.00569	0.00903	0.06433	0.01299
33	0.07707	0.01254	0.00335	0.00530	0.06233	0.01006	0.10065	0.01763	0.00555	0.00841	0.07128	0.01381
35	0.08466	0.01454	0.00321	0.00498	0.06862	0.01148	0.11072	0.01985	0.00548	0.00786	0.07839	0.01510

**Table 2:** Comparison of average number of iterations of matched pixels in ROI_1_, ROI_2_, and ROI_1&2_ with different convergence thresholds for IC-GN_1_ and IC-GN_2_.

Threshold for $\left\|\Delta P_{main}\right\|$	${\bar{n}}_{itor}$-ROI_1_		${\bar{n}}_{itor}$-ROI_2_		${\bar{n}}_{itor}$-ROI_1&2_	
	IC-GN_1_	IC-GN_2_	IC-GN_1_	IC-GN_2_	IC-GN_1_	IC-GN_2_
0.1	1.0110	1.4293	1.0024	1.3989	1.0063	1.4141
0.01	1.4927	2.4875	1.3874	2.4457	1.4401	2.4666
0.001	2.4787	3.8182	2.3830	3.7693	2.4308	3.7937
0.0001	3.6110	5.1762	3.5212	5.1098	3.5661	5.1430

**Table 3:** Plane and cylinder surface fitting results of 3D coordinates measured by CMM.

	Point Number	Standard Deviation	Positive Maximum	Negative Maximum
Plane	15	0.001 mm	0.003 mm	−0.004 mm
Cylinder	44	0.004 mm	0.011 mm	−0.008 mm

**Table 4:** Statistics of matching rates with different subset sizes measured by IC-GN_1_ and IC-GN_2_.

SS	Plane			Cylinder			Back of Head		
	$N_{pix}$	$r_{m}$ (%)		$N_{pix}$	$r_{m}$ (%)		$N_{pix}$	$r_{m}$ (%)	
		IC-GN_1_	IC-GN_2_		IC-GN_1_	IC-GN_2_		IC-GN_1_	IC-GN_2_
15	90,000	99.93	99.84	90,000	100	99.01	62,500	63.95	98.77
17	90,000	99.99	99.97	90,000	100	99.29	62,500	64.52	98.93
19	90,000	100	99.97	90,000	100	99.31	62,500	65.09	99.00
21	90,000	100	99.98	90,000	100	99.35	62,500	65.55	98.99
23	90,000	100	99.98	90,000	100	99.38	62,500	65.62	98.99
25	90,000	100	99.98	90,000	100	99.38	62,500	66.27	98.98
27	90,000	100	99.98	90,000	100	99.38	62,500	67.01	99.01
29	90,000	100	99.98	90,000	100	99.35	62,500	67.71	98.99
31	90,000	100	99.98	90,000	100	99.40	62,500	68.15	98.97
33	90,000	100	99.98	90,000	100	99.41	62,500	68.60	98.89
35	90,000	100	99.98	90,000	100	99.38	62,500	68.80	98.88
